# Supplementary material for: The discovery of novel noncoding RNAs in 50 bacterial genomes
Source: Nucleic Acids Res. 2024 Apr 22;52(9):5152–65. doi: 10.1093/nar/gkae248 (PMC11109978; doi:10.1093/nar/gkae248)
Supplement: gkae248_Supplemental_Files [file gkae248_supplemental_files.zip › Narunsky 50 Genomes RNA Motifs Files.zip!/Publication/69-E_rhusiopathiae/Figures/LRC-69-24-NC_015601_1_IGR_69_1198638_1198886.sto.pdf]

LRC-69-24-NC_015601_1_IGR_69_1198638_1198886.sto.r2r

5′-AUAUUUUAUCCCUCG●YCYUAUUAUAAC●AAAA●YYA●GUGAAUGCUAGUGUGAAAUUAUCG●AUUU●UAC●C●CAAUCAUAY●YYC●C○○○○RC●AUU●AGACUUUUYAAUCUCA●AUUYRA●CYUGAYACACUU●UGU●YCAC●G●YAAYAAC●YACU●●C●R●R●CC●●UCUYRCACAA●GCGRARCY●AA○○○○○A●●AYACA●●●GAAUG○○CAAY●●Y●RA●UGA●ACGAAAC●AUCUCA○○
